# Automatic identification of gait events using an instrumented sock

**DOI:** 10.1186/1743-0003-8-32

**Published:** 2011-05-27

**Authors:** Stephen J Preece, Laurence PJ Kenney, Matthew J Major, Tilak Dias, Edward Lay, Bosco T Fernandes

**Affiliations:** 1 Centre for Health, Sport and Rehabilitation Sciences Research, Blatchford Building, University of Salford, Manchester, M6 6PU, UK; 2 School of Art and Design, Nottingham Trent University, Burton Street, Nottingham, Nottinghamshire, NG1 4BU, UK; 3 School of Materials, The University of Manchester, Manchester, M13 9PL, UK; 4 School of Electrical and Electronic Engineering, The University of Manchester, Manchester, M13 9PL, UK

## Abstract

**Background:**

Textile-based transducers are an emerging technology in which piezo-resistive properties of materials are used to measure an applied strain. By incorporating these sensors into a sock, this technology offers the potential to detect critical events during the stance phase of the gait cycle. This could prove useful in several applications, such as functional electrical stimulation (FES) systems to assist gait.

**Methods:**

We investigated the output of a knitted resistive strain sensor during walking and sought to determine the degree of similarity between the sensor output and the ankle angle in the sagittal plane. In addition, we investigated whether it would be possible to predict three key gait events, heel strike, heel lift and toe off, with a relatively straight-forward algorithm. This worked by predicting gait events to occur at fixed time offsets from specific peaks in the sensor signal.

**Results:**

Our results showed that, for all subjects, the sensor output exhibited the same general characteristics as the ankle joint angle. However, there were large between-subjects differences in the degree of similarity between the two curves. Despite this variability, it was possible to accurately predict gait events using a simple algorithm. This algorithm displayed high levels of trial-to-trial repeatability.

**Conclusions:**

This study demonstrates the potential of using textile-based transducers in future devices that provide active gait assistance.

## Background

Foot drop is currently estimated to affect approximately 20% of stroke survivors [1]. With this condition, patients are unable to dorsiflex their ankle due to weak dorsiflexor muscles, often accompanied by shortening, contracture and/or spasticity of the plantarflexors [2]. In the absence of compensatory movements, such as hip circumduction, the foot can fail to clear the ground during the swing phase of gait and can often land in an inappropriate orientation [3]. As a result, foot drop gait is slow and energy inefficient and likely associated with an increased fall risk [4-6].

There are a number of assistive devices which are designed to minimise the effect of foot drop by maintaining the foot in appropriate orientation during gait. These approaches can be considered as either passive devices, such as ankle foot orthoses, or active devices, such as functional electrical stimulation (FES) [7] or intelligent orthoses [8,9]. FES for foot drop conventionally involves stimulation of the peroneal nerve during gait to dorsiflex the foot and is now supported by a substantial body of evidence [10-12]. However, in order to stimulate muscular contraction during the appropriate period of gait, FES and other active systems require precise information on gait phase.

Most current FES systems obtain gait phase information from a footswitch located in the heel region of the shoe. Although, with appropriate signal conditioning, accurate detection of heel strike and heel lift is possible, footswitches can be time consuming to set up, are prone to false event detections when the user weight shifts and reports have suggest that users dislike them [13]. Further, recent studies have demonstrated the benefits of additionally stimulating the plantarflexor muscles during the terminal double-support phase of gait, requiring the use of 2 footswitches in each shoe [14]. In some systems a connecting wire is required from the shoe to the stimulator which can be cumbersome to users. Furthermore, as the footswitch must be consistently located relative to the foot, shoes must be worn and so this approach is not well suited to indoor use. Inertial sensors have been suggested as an alterative to footswitches for detecting gait phase [15,16]. However, this approach, which typically relies on inferring gait events from motion of the shank, can be problematic for users with particularly poor gait. Furthermore, neither footswitches nor inertial sensors provide a direct measurement of ankle joint motion.

Anecdotal reports suggest that, despite training, a number of users of conventional FES devices locate their electrodes incorrectly, resulting in a poor foot response during gait. With the advent of electrode array-based stimulation systems [17,18] that allow for the software control of both the location and intensity of stimulation, there is the potential to automate both the set up process and cycle-cycle control of stimulation. Such an approach would require a method of monitoring of gait phase and foot orientation, not available from footswitch data.

An alternative to either footswitches or inertial sensors is to derive information on gait phase from a sock incorporating a textile-based transducer. This approach offers a number of advantages. Firstly, it would be possible to integrate the stimulation unit into the sock and therefore eliminate the need for connecting wires and increase the ease of use to the patient. Secondly, the system would work without footwear and so would be more suited to indoor use. Finally, as the sensor measures the joint motion itself, rather than inferring events from foot loading or shank motion, it may be possible to obtain more detailed information on gait.

Textile-based transducers consist of either yarns made from conductive elastic fibers or conductive coatings applied to elastic base materials. Both approaches utilise piezo-resistive properties to sense strain. Most previous work in this area has focused on either knitted fibre transducers [19] or smeared conductive elastomers [20-24]. With the first approach, conductive fibers are knitted within a non-conducting base material, whereas with the latter approach a mixture of conductor and flexible material is smeared onto a flexible substrate. To date, textile-based transducers have been successfully utilised for hand posture recognition [20], classification of upper limb gestures [21] and postures [23,24], monitoring respiratory rate [19,25] and detecting events in a knee flexion trajectory during a landing movement [26]. However, there has been no previous work attempting to derive information on gait phase from a sensor positioned at the ankle.

Textile-based transducers exhibit a high degree of non-linearity in the relationship between resistance and deformation. One of the primary causes of this non-linearity is the viscoelatic properties of the textiles which results in a number of phenomena, such as velocity dependent resistance peaks, delayed recovery after rapid stretching and hysteresis. In previous applications, these effects have been overcome using either complex mathematical models [21] or machine learning algorithms [23]. However, in walking, gait phase information can be obtained from ankle motion in the sagittal plane, which undergoes periods of rapid movement followed by periods of relatively slow change. Given the nature of this movement, we wanted to investigate whether it would be possible to extract the salient features of ankle motion, and therefore derive information on gait phase, without using a complex modelling approach. This would clearly be advantageous in any embedded system designed to trigger FES system during walking. In order to address our research objective, we sought to answer two questions. Firstly, we aimed to investigate the degree to which the output of the instrumented sock matched the ankle joint angle for a range of individuals, under sock-only and shod conditions. Secondly, we aimed to establish whether it would be possible to identify the three key gait events, heel lift (HL), toe off (TO) and heel strike (HS) using a relatively straight-forward prediction algorithm. It was felt that this proof of concept study was necessary to assess the suitability of a textile-based transducer for use with FES systems and other future active gait assistive devices.

## Methods

### A Wearable Sensor Technology

**Figure 1:**
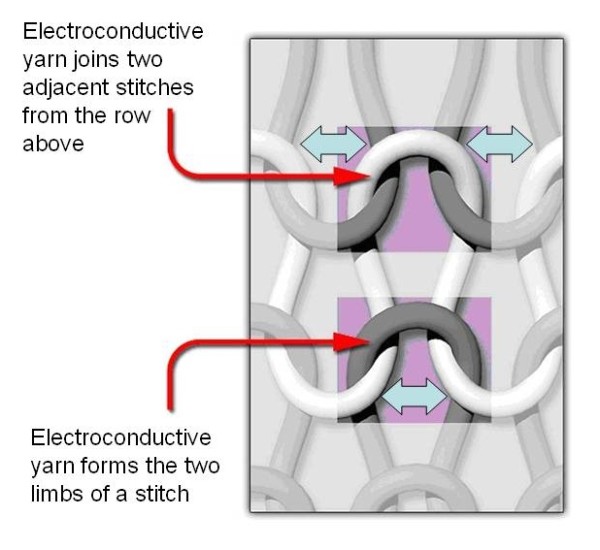
**Structure of the resistive strain sensor**. The individual stitches which make up the knitted resistive strain sensor. The electroconductive yarn is shown in white and arrows mark the regions where electrical contact is made as the base structure relaxes after stretching.

The textile-based transducer investigated in this study was a knitted resistive strain sensor. Knitted structures consist of stitches which are arranged in rows and columns and which are bound to the stitches above and below as shown in Figure 1. The sensor under investigation is knitted from a non-conductive elastomeric base material with a low modulus of elasticity. Within this base structure, a predetermined segment of one row of stitches is knitted from an electroconductive yarn (Figure 1). This yarn consisted of Nylon 6.6 filaments, coated with a nano layer of silver. During the knitting process the textile structure is subjected to a high degree of stretch, after which the base structure relaxes, drawing the stitches together. This creates contact between adjacent parts of the electroconductive yarn in the regions where the electroconductive yarn forms the two limbs of a stitch (Figure 1). This contact reduces the effective conductive length of the yarn, lowering the electrical resistivity. However, stretching the knitted structure widthways has the effect of breaking the contact between adjacent stitch limbs and therefore increasing electrical resistivity. This resistive strain sensor technology is patented by SmartLife Technologies Ltd and was incorporated into a knitted sock by the knitting research group at the University of Manchester. For the sock, the electroconductive yarn was knitted into two parallel rows of stitches, connected at the toe end of the sock. With this design electrical connectors where placed at the other end of the sock.

**Figure 2:**
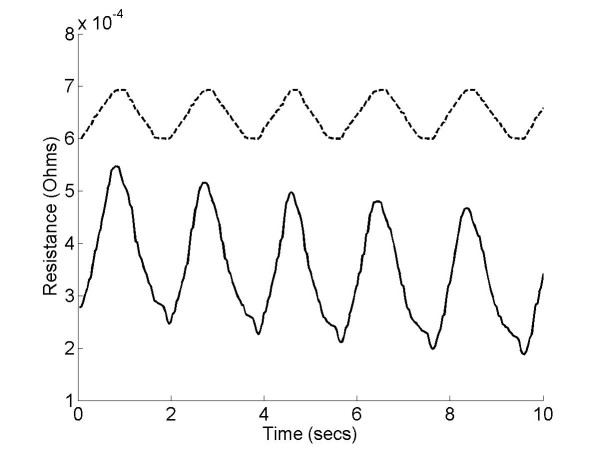
**Resistive properties of the instrument sock**. Plot of resistance (solid line) against time showing how the knitted sensor responds to a periodically applied strain (dotted line). This data shows a gradual drift in the baseline resistance after several successive stretches.

**Figure 3:**
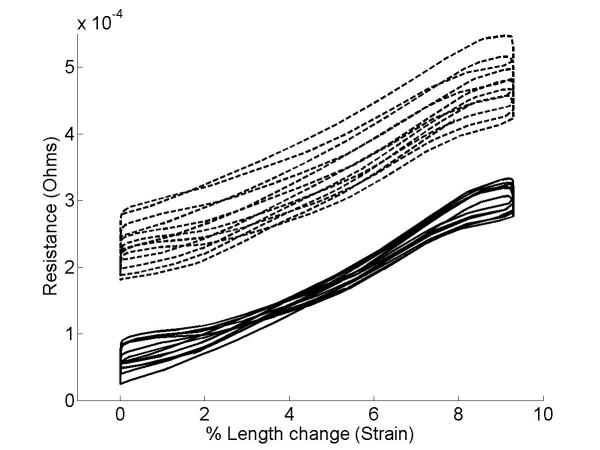
**Resistive properties of the instrument sock**. Plot of resistance against length change over several stretch-relaxation cycles (dotted line) and the same data after high pass filtering of the resistance data (solid line).

In order to understand how the resistance of the knitted sensor changed in response to an applied strain, we measured the resistance of a sample undergoing repeated stretching and relaxing. Figure 2 shows how the resistance varies over time when the sample is repeatedly stretched and relaxed at 9 mm per second. From this plot it is clear that the baseline resistance of the sample gradually decreases over time, however further analysis showed that this drift could largely be eliminated by high pass filtering the data at 0.3 Hz. Figure 3 shows a plot of resistance against strain before and after high pass filtering. Although there is some degree of hysteresis, most likely due to the visco-elastic properties of the textile structure, removal of the baseline drift produces an approximately linear relationship between strain and resistance.

### B Data collection

**Figure 4:**
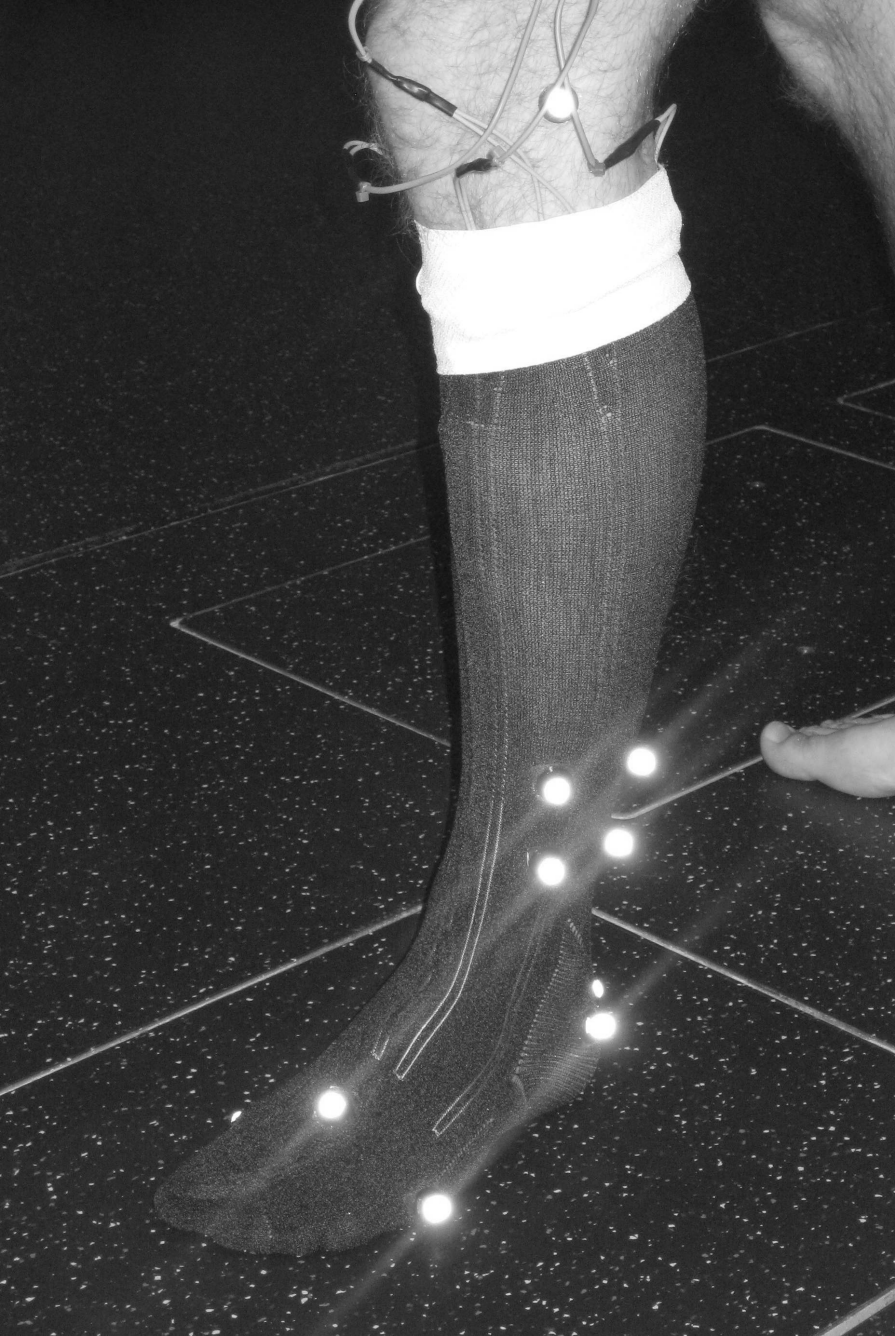
**Experimental set up**. Image of the instrumented sock with the kinematic markers used for data collection during the sock-only walking trials.

For the main experimental work each subject wore an instrumented sock on their left leg (Figure 4). To ensure that a similar fit was obtained for all subjects we selected between five different sizes of sock depending on the length and maximum circumference of each subject's shank. The sock was secured at the proximal end with an overwrapped bandage and the sensor connected to a constant current source power supply. The output of this set up (which was proportional to the sensor resistance) was then fed into a Noraxon Telemyo 2400 T G2 data transmission unit. This unit digitised the input voltage, which typically ranged from 0.2-0.4 V peak-to-peak, at a sampling frequency of 1500 Hz. The digitised data was then transmitted to a laptop for visual checking and storage.

In order to derive kinematic signals during walking, 3D data from a number of reflective markers (Figure 4) were collected using a ten-camera Qualisys Pro Reflex system operating at 100 Hz. Calibration markers were placed on the femoral epicondyles, the ankle malleoli and the 1^st ^and 5^th ^metatarsal heads. In addition, tracking markers were placed on the lateral aspect of the shank, calcaneous and dorsal aspect of the midfoot. Although previous studies have recommended using a shank marker plate with underwrapped bandage [27], pilot work showed us that a bandage could interfere with the sock output signal. Therefore markers were fixed directly to the sock with adhesive tape. A static calibration trial was collected for each condition (sock-only and shod) after which the calibration markers where removed for the main walking trials.

Twenty subjects (eight female) were recruited into the study. The mean (SD) age of the subjects was 43 (18), mean (SD) height 171 (8) cm and mean (SD) weight 72 (12) Kg. Each subject provided written consent to participate and ethical approval was granted by the institutional ethics committee. Each subject performed ten walking trials, at their self selected walking speed, in both a sock-only condition and in a shod condition. Each trial consisted of approximately 15 steps, with trials being separated by approximately 40 seconds. The sock was not removed between the different trials and for the shod condition subjects wore their normal shoes. Synchronised kinematic and digitised sock voltage data was collected for each set of ten trials. Although gait event timings from consecutive gait cycles can be collected using footswitches, they can only be used during shod gait. For this study we wanted to investigate sensor output in both a sock-only and shod condition. Therefore force-plate data from two AMTI force platforms was used to collect kinetic data, allowing for identification of a single gait cycle for each trial.

### C Data Processing

Kinematic data was processed by using the static calibration to calculate ankle joint centre and define segmental coordinate systems for the shank and foot. The 3D coordinate data for each trial was then used to calculate using Cardan angles. All kinematic calculations were implemented using Visual3D (C-Motion Inc) and the data for both sets of trials for all twenty subjects exported to Matlab for further processing.

In order to compare the kinematic data with the sensor data, the kinematic data was upsampled to 1500 Hz, matching the collection frequency of the sensor data. Two consecutive heel strikes were then identified from the two force platforms as the point at which the vertical component of the ground reaction force exceeded 5N. These points were then used to define the gait cycle data for both the kinematic and the sensor signal. HL was then identified as the minimum in the kinematic signal occurring just before toe off. In order to locate this minimum, the raw 3D coordinate data was low pass filtered at 6 Hz (zero lag 4^th ^order Butterworth filter) to remove measurement noise. The minimum in the kinematic signal corresponds to the point at which the ankle begins to plantarflex in preparation for toe off.

**Figure 5:**
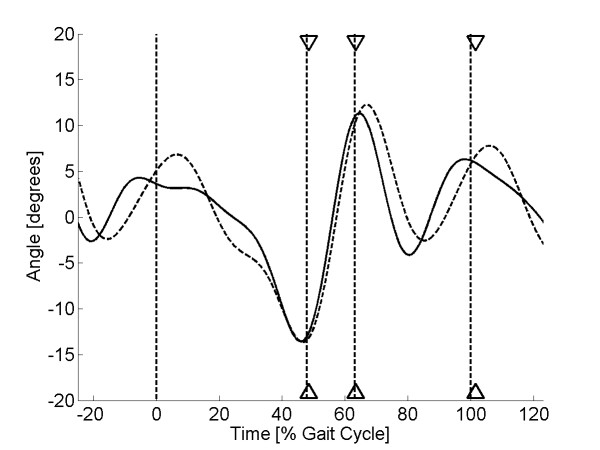
**Sensor output (sock-only condition) for subject 1**. Plot of sensor output (solid line) and scaled kinematic signal (dashed line) against time for a single walking trial from subject 1 (sock-only condition). The three sets of triangles show the estimated times of heel lift, toe off and heel strike with the vertical dashed lines showing the true values.

**Figure 6:**
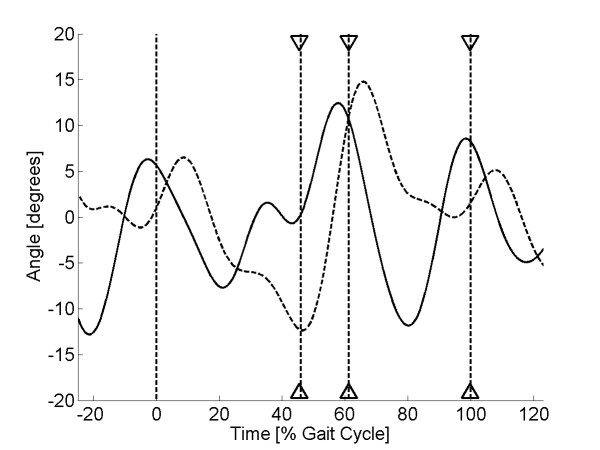
**Sensor output (sock-only condition) for subject 20**. Plot of sensor output (solid line) and scaled kinematic signal (dashed line) against time for a single walking trial from subject 20 (sock-only condition). The three sets of triangles show the estimated times of heel lift, toe off and heel strike with the vertical dashed lines showing the true values.

**Figure 7:**
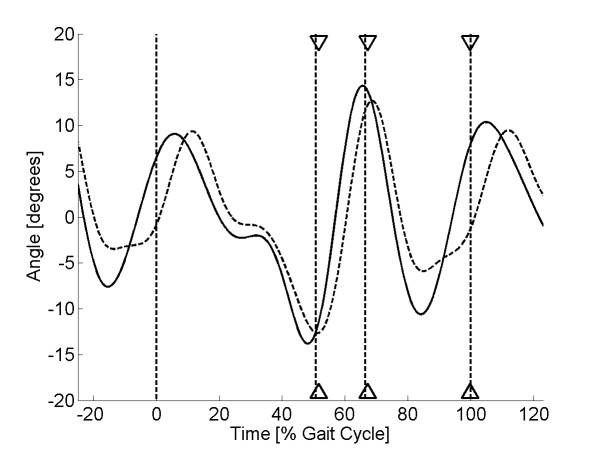
**Sensor output (shod condition) for subject 8**. Plot of sensor output (solid line) and scaled kinematic signal (dashed line) against time for a single walking trial from subject 8 (shod condition). The three sets of triangles show the estimated times of heel lift, toe off and heel strike with the vertical dashed lines showing the true values.

**Figure 8:**
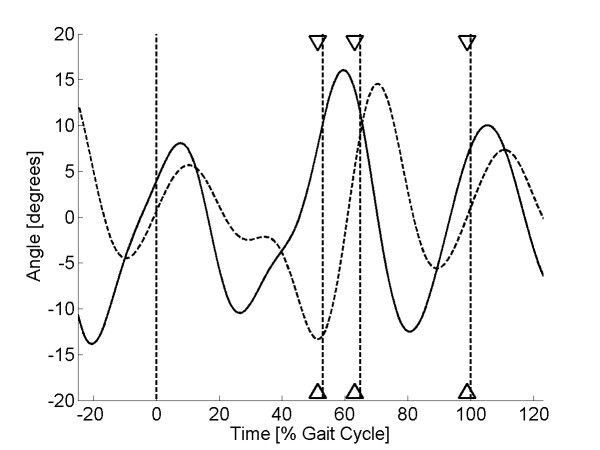
**Sensor output (shod condition) for subject 9**. Plot of sensor output (solid line) and scaled kinematic signal (dashed line) against time for a single walking trial from subject 9 (sock-only condition). The three sets of triangles show the estimated times of heel lift, toe off and heel strike with the vertical dashed lines showing the true values.

As discussed earlier, high pass filtering of the sensor signal at 0.3 Hz was required to remove the baseline drift in the sensor output. This frequency was chosen as the best compromise to remove the baseline drift in the sensor signal, yet still retain the low-frequency component of human walking. Pilot investigation showed that optimal gait event recognition could be obtained when the sensor signal was low pass filtered at 4 Hz. Therefore, band pass filtering (0.3-4 Hz) was applied to both the sensor and the kinematic signal using a FFT filter. This allowed the variation between the two signals to be compared, irrespective of the signal means. Finally, to remove the effects of signal amplitude, the filtered sensor signal was scaled so that the peak-to-peak range matched that of the filtered kinematic signal. Example plots for two sock-only and two shod trials are shown in Figures 5, 6, 7 &8.

In order to address our first research question, which was aimed at understanding the match between the kinematic and sensor signal, we used two separate measures to quantify signal similarity. These measures have been used previously to evaluate the accuracy of wearable sensors in the prediction of lower limb kinematics [28] and are given as:

1. Pearson's correlation coefficient, r.

2. The normalised mean absolute deviation, , where  and  are the *i*th angle and *i*th voltage data points in the kinematic and sensor curves after both have been scaled to have a peak-to-peak range of unity. The number of data points across the whole gait cycle is given as *n*.

Separate measures of signal similarity were obtained for the sock-only and shod conditions by averaging across the ten gait cycles (one from each walking trial).

For this proof of concept study we aimed to investigate whether a relatively simple algorithm could be used to identify the three gait events from the sensor signal. Although sensor data from each subject displayed similar features, these features occurred at different points in the gait cycle. It was therefore necessary to adjust the algorithm parameters separately for each individual subject. Our data showed that sensor output could sometimes be modified when shoes were worn. Therefore, algorithm parameters were adjusted separately for the sock-only and the shod conditions. As our dataset consisted of a single gait cycle for each trial, the algorithm was implemented as a forward search, staring from *t = 0 *(the first HS), to determine the time of HL, TO and the second HS. Although this was tested on the ten separate trials, it could potentially be implemented on a continuous sensor signal to identify consecutive gait events.

**Figure 9:**
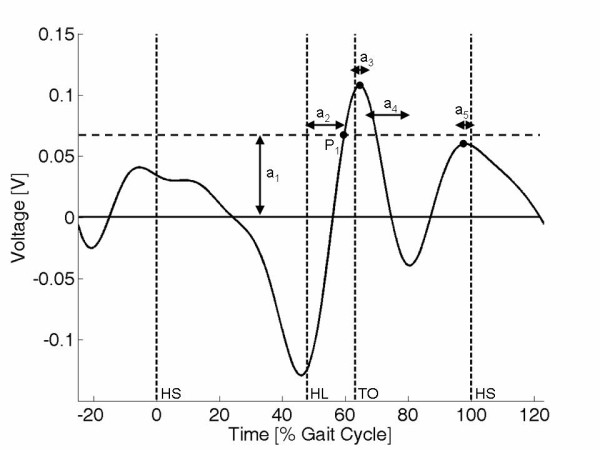
**The event detection algorithm**. Plot to illustrate the five parameters used in the event detection algorithm.

The final algorithm operated using the stages outlined below and required adjustment of five parameters (*a_1_-a_5_*). Each of these parameters is depicted in Figure 9. Note that this plot has the units of voltage on the y-axis as it operated on the raw data from the sensor signal. The three gait events were identified from the sensor signal as follows:

1. Identify the first point (P_1_) where the sensor signal is increasing and exceeds a preset threshold (*a_1_*). HL was then identified to be a fixed time offset (*a_2_*) from this point.

2. Find the first maxima after P_1_. TO was then identified to be a fixed time offset (*a_3_*) from this maxima.

3. Advance by a fixed time (*a_4_*) then find the next maxima. HS was then identified to be a fixed time offset (*a_5_*) from this maxima.

The five parameters (*a_1_-a_5_*). were obtained from the first five trials of each subject/condition using an automated search algorithm. This analysed the maximal values of the signal over the initial stages of the gait cycle in order to determine the threshold *a_1_*. It then calculated the mean values of *a_2_-a_5 _*required to accurately identify the three gait events. To ensure that the algorithm would work effectively with a raw voltage signal the four time offsets (*a_2_-a_5_*) were fixed in seconds, rather that gait cycle time. Once the values for the five parameters had been set using data from the first five trials, they were used to predict the three gait events for the final five trials. Algorithm accuracy was then calculated as the mean absolute deviation (in %gait cycle) between the predicted time and true time across the five trials. In addition, the standard deviation of the difference between the true and predicted time (%gait cycle) was used to capture the trial-to-trial repeatability in event prediction.

## Results

Visual inspection of the sensor curves showed that they displayed the same general characteristics as the kinematic signals for both the sock-only and shod conditions (Figures 5, 6, 7 &8). Specific characteristics included maxima around HS and TO and minima around HL and between TO and HS. However, although data for some subjects showed a close match between the two conditions, high correlations and low mean absolute differences (nMAD), data from other subjects was markedly different (Table 1). To illustrate these differences, kinematic and sensor signals for a single trial have been plotted for subjects 1 and 20 who showed the best and the worst match respectively for the sock-only condition (Figures 5 and 6). Similar data has been shown for the shod conditions for subject 8 (best match) and 9 (worst match) in Figures 7 and 8.

**Table 1:** Comparison between the kinematic and sensor signals

	Sock-only		Shod	
**Subject**	**r**	**nMAD**	**r**	**nMAD**

1	0.91	0.09	0.59	0.23

2	0.77	0.16	0.56	0.22

3	0.84	0.13	0.7	0.18

4	0.91	0.1	0.84	0.12

5	0.79	0.16	0.39	0.27

6	0.92	0.1	0.87	0.12

7	0.85	0.14	0.83	0.13

8	0.92	0.09	0.8	0.15

9	0.65	0.16	0.05	0.29

10	0.83	0.14	0.7	0.17

11	0.75	0.17	0.4	0.26

12	0.74	0.17	0.5	0.25

13	0.91	0.1	0.71	0.19

14	0.8	0.16	0.56	0.23

15	0.76	0.16	0.82	0.13

16	0.86	0.13	0.5	0.23

17	0.66	0.2	0.53	0.23

18	0.71	0.19	0.18	0.28

19	0.76	0.16	0.46	0.23

20	0.28	0.26	0.22	0.26

Mean	0.78	0.15	0.56	0.21

Two measures of similarity between the filtered sensor signal and the filtered and scaled kinematic signal for the sock-only and the shod condition. The measures are the correlation coefficient (r) and the normalised mean absolute difference (nMAD).

The algorithm developed to predict gait events was found to be accurate for HL and TO for both sock-only and shod conditions with mean errors across subjects ranging from 1-1.6% gait cycle (Tables 2 and 3). Errors for HS were slightly higher for both conditions (means 2.6 & 3.3% gait cycle) but still within an acceptable accuracy (Tables 2 and 3). Standard deviations for each of the three gait events were comparable with the mean errors for both the sock-only (Table 2) and shod condition (Table 3), demonstrating good trial-to-trial repeatability in event prediction. On Figures 5, 6, 7 &8 we have illustrated the true and predicted gait events for a number of different signals. These plots show that, even when there was a mismatch between the kinematic and sensor signal, the algorithm was still able to predict gait events to a high level of accuracy. Although it was possible to implement the prediction algorithm successfully for all subjects in the sock-only condition, it could not be applied to one subject in the shod condition. This was due to a peak at approximately 30% gait cycle which was similar in magnitude to the peak at TO and which often exceeded the preset threshold (*a_1_*). For these data a more complex algorithm would be required.

**Table 2:** Gait prediction error for the sock-only condition

Subject	Mean HL	Std HL	Mean TO	Std TO	Mean HS	Std HS
1	0.6	0.2	0.4	0.5	0.9	1.2

2	0.6	0.6	0.3	0.2	0.8	0.5

3	0.6	0.8	0.7	0.7	2.6	0.7

4	1.6	0.8	0.8	0.2	2.8	3.1

5	2.1	1.7	0.5	0.5	1.8	1.9

6	0.2	0.3	0.5	0.2	2.1	1.9

7	1.6	0.8	0.9	1.4	4.5	3

8	0.6	0.6	0.7	0.7	2.6	0.6

9	1.8	0.8	1.8	2	1.3	1.3

10	1.6	2	2.3	2.5	3.5	6.4

11	0.7	0.8	1.1	0.9	3.8	1

12	1.4	1.9	1.8	2.8	5.1	7.7

13	0.5	0.6	0.3	0.4	3.3	1.9

14	0.9	0.4	0.3	0.4	2.4	1.5

15	2.2	1.6	0.9	1.1	2	2.3

16	1.9	1.7	1.6	2.5	2.6	3.6

17	2.3	2.2	1.5	1.6	1.2	1.4

18	1.9	2.4	1.5	1.8	3.6	5.2

19	1.2	1	0.6	0.7	4	2.3

20	1.3	1.2	1.5	1.5	0.9	1

Average	1.3	1.1	1	1.1	2.6	2.4

Mean and standard deviation of the error in the prediction of the three gait events, HL (heel lift), TO (toe off) and HS (heel strike), expressed as %Gait cycle for sock-only condition.

**Table 3:** Gait prediction error for the shod condition

Subject	Mean HL	Std HL	Mean TO	Std TO	Mean HS	Std HS
1	-	-	-	-	-	-

2	0.5	0.4	0.6	0.7	0.8	1

3	0.8	0.9	0.6	0.6	2.1	2.5

4	0.7	1	0.4	0.4	4	3.8

5	3.7	3.6	8.6	7.2	15.2	17.1

6	0.8	1	0.3	0.3	5.7	6.5

7	1.6	1.1	1.1	1.5	1.7	1.7

8	1	1	1.8	1.2	0.4	0.5

9	1.2	1.5	0.9	0.9	3.1	2.3

10	0.8	0.7	1.1	1.1	1.4	1.5

11	1.3	1.2	0.8	0.7	1.7	1.6

12	4.5	4.4	1.8	2.7	6.1	9.8

13	3	2.4	1.2	0.4	1	0.9

14	1	0.8	0.7	0.8	1.2	1.4

15	0.8	1.1	0.8	0.8	2.7	1

16	3.3	2	2.4	3.3	4.1	3.5

17	1.2	1.7	0.4	0.4	0.3	0.4

18	2.3	2.4	2.9	4.6	4.3	7.2

19	1	1.4	0.8	0.9	5.9	7

20	1.2	2	1	0.9	0.8	0.8

Average	1.6	1.6	1.5	1.6	3.3	3.7

Mean and standard deviation of the error in the prediction of the three gait events, HL (heel lift), TO (toe off) and HS (heel strike), expressed as %Gait cycle for the shod condition.

## Discussion

This study was designed to establish the possibility of using a textile-based transducer to extract the salient features of ankle joint motion and derive information on gait phase during walking. The results demonstrated that the output of the sensor displayed the same features as the ankle joint kinematic signal. However, the exact match between these two signals varied considerably between individuals. Despite this variability, it was possible to accurately predict gait events using a simple algorithm which also showed good levels of trial-to-trial repeatability.

The textile-based transducer examined in this study exhibited a number of nonlinear characteristics. Although it was possible to remove the effect of baseline drift using high pass filtering, preliminary characterisation demonstrated hysteresis in the relationship between resistance and strain. Despite this non-linearity, data from some subjects demonstrated a very close match between sensor output and the ankle joint kinematic signal (Figures 5 &7). However, in other subjects there were large discrepancies between the two signals (Figures 6 &8). It is possible that these between-subject differences were the result of differences in the fit of the sock which could have resulted in the knitted structure operating around a different point in the resistance-strain curve. A possible future approach to overcoming this problem would be to produce a bespoke sock for each subject to ensure that the amount of strain experienced by the sensor does not differ greatly between subjects. Alternatively, it may be possible to use a more complex modelling approach to predict the response of the sensor across different individuals. However, although modelling approaches have been used before in studies of textile-based transducers [21], they may not be a viable option for an embedded FES controlled which must work in real-time.

To investigate the possibility of using a textile-based transducer in future FES applications, we developed an algorithm for gait event detection which was based around two specific signal features. These were a rapid increase and peak around TO and a subsequent peak at the end of the gait cycle. The first of these two features corresponds to the rapid ankle plantarflexion which occurs just prior to TO. Our analysis showed that this feature exhibited high levels of step-to-step repeatability as demonstrated by the low standard deviations in the prediction accuracy of HL and TO. However, the larger standard deviations found for HS showed that the second feature, the peak at the end of the gait cycle, exhibited slightly lower levels of step-to-step repeatability.

Previous studies have investigated the accuracy of using footswitches, accelerometers, gyroscopes and even neural sensors [29] to predict gait events. Footswitches are used in most commercial FES applications and have been shown to predict gait events to within 0.5-2% gait cycle [30,31], slightly better than the accuracies reported in this study. In a recent study, Lau and Tong [16] investigated the potential of using accelerometers and gyroscopes to identify gait events in both healthy subjects and subjects with foot drop. Using an approach similar to that presented in this paper, they investigated the step-to-step variability in timing of maxima and minima in the sensor signals, suggesting these points could be used as the basis of a gait event prediction algorithm. Their results showed that, in healthy subjects, peaks in accelerometer signals mounted on the foot or shank, showed a mean variability of approximately 2% gait cycle, similar to the accuracies reported in this paper. Mansfield and Lyons [32] investigated the possibility of using a trunk-mounted accelerometer to identify heel contact of both limbs. Their study showed that there was an observable delay between heel contact and a negative-positive change in acceleration. However, most subjects demonstrated a relatively large standard deviation in this delay which equated to approximately 2-8% gait cycle. Sinkjaer et al [33] reviewed the small number of studies reporting on the use of neural sensor signals for detecting heel strike and foot lift off. When used in conjunction with a machine learning algorithm in a subject with foot drop, these signals were shown to provide detection of heel strike within 50 ms. However, detection of TO tended to be less accurate with errors up to 220 ms, equating to more than 10% of the gait cycle.

There are a number of limitations to the current proof of concept which will need to be addressed if systems using textile-based transducers are to be used in future FES or other active gait assist devices. Firstly, the proposed algorithm locates HL and TO at specific times behind the occurrence of events in the sensor signal. This means that, although the algorithm works well for off-line processing, it would not be effective in a real-time system. Furthermore, the proposed algorithm requires a number of individual-specific thresholds to be set. Future work must now focus on algorithms which can automatically adapt to individual differences in sensor output and predict gait events from signal characteristics which occur before the required gait events. With more advanced approaches it should be possible to eliminate the need for manual adjustment of thresholds whilst still maintaining a level of computational complexity which could be implemented within an embedded controller. Another limitation of the study was that it was performed on individuals with normal gait patterns in a controlled laboratory environment. Clearly, future work must focus on patients with drop foot and establish the feasibility of using an instrumented sock in a real-world setting.

## Conclusions

In summary, our data showed considerable inter-subject variability in the match between the signal from an instrumented sock and ankle motion in the sagittal plane during normal walking. However, using a relatively straight-forward algorithm, we were able to predict three gait events to a high degree of accuracy with good trial-to-trial repeatability. Although more complex algorithms would be required, our results demonstrate the potential of using a textile-based transducers in future FES applications.

## Competing interests

The authors declare that they have no competing interests.

## Authors' contributions

SJP was involved in the design of the study, data collection and writing of the manuscript. LPJK and TD conceived the original idea, contributed to the study design and helped to draft the manuscript. MJM was involved in data collection, processing and in some aspects of the experimental design and EL and BF were involved in the development of the experimental set up including the design and manufacture of the instrumented socks All authors read and approved the final manuscript.
